# 赛沃替尼诱导*MET*扩增非小细胞肺癌病理学完全缓解1例

**DOI:** 10.3779/j.issn.1009-3419.2024.102.36

**Published:** 2024-11-20

**Authors:** Meng LU, Ran ZHANG, Baiwei LI, Haidi XU, Yongkuan GUO, Jian YOU, Bingsheng SUN

**Affiliations:** ^1^300000 天津，天津医科大学肿瘤医院肺部肿瘤科，国家恶性肿瘤临床医学研究中心，天津市恶性肿瘤临床医学研究中心（鲁蒙，尤健，孙冰生）; ^1^Department of Pulmonary Oncology, Tianjin Medical University Cancer Institute & Hospital, National Clinical Research Center for Cancer, Tianjin’s Clinical Research Center for Cancer; ^2^天津市肿瘤医院空港医院胸部肿瘤科，国家恶性肿瘤临床医学研究中心（张然，李百玮，徐海迪，郭永宽）; ^2^Department of Thoracic Oncology, Tianjin Cancer Hospital Airport Hospital, National Clinical Research Center for Cancer, Tianjin 300000, China

**Keywords:** 肺肿瘤, 新辅助靶向治疗, MET扩增, 赛沃替尼, 病理学完全缓解, Lung neoplasms, Neoadjuvant targeted therapy, MET amplification, Savolitinib, Pathological complete response

## Abstract

间质-上皮细胞转化因子（mesenchymal-epithelial transition factor, MET）基因突变是非小细胞肺癌（non-small cell lung cancer, NSCLC）中常见的一类基因突变，包括MET外显子14跳跃突变（MET exon 14 skipping mutation, METex14m）和MET扩增（MET amplification, METamp）等类型。针对具有METex14m的晚期NSCLC，赛沃替尼（Savolitinib）这种酪氨酸激酶抑制剂（tyrosine kinase inhibitors, TKIs）具有较高的敏感性。METamp是一种较为罕见的NSCLC中的基因突变类型，可以作为驱动基因介导表皮生长因子受体（epidermal growth factor receptor, EGFR）-TKIs的原发性耐药和后期药物获得性耐药。对于EGFR-TKIs诱导产生的继发性METamp突变晚期NSCLC，临床多采取在EGFR-TKIs基础上联合MET-TKIs进行治疗，而针对原发性METamp突变晚期NSCLC的最佳治疗策略尚未确定。对于EGFR、间变性淋巴瘤激酶（anaplastic lymphoma kinase, ALK）融合、METex14m等驱动基因突变阳性的局部晚期NSCLC患者，通过新辅助靶向治疗获得良好预后已有相关病例报道，但目前尚未有METamp突变的局部晚期NSCLC患者进行新辅助靶向治疗的案例。本文报道了1例存在双驱动基因突变（EGFR L858R合并原发METamp）的局部晚期NSCLC患者，经过吉非替尼单药治疗1个月后肿瘤未见缩小，更换为赛沃替尼单药治疗4个月诱导后肿瘤明显消退，根治性手术切除后病理结果提示肿瘤获得病理学完全缓解，术后患者对持续赛沃替尼治疗反应良好，至今未见肿瘤复发或转移。本文首次报道了原发METamp突变的局部晚期NSCLC患者行新辅助靶向治疗的可行性和有效性，以期为原发METamp突变局部晚期NSCLC的围手术期治疗提供有效借鉴。

## Abstract (en)

Mesenchymal-epithelial transition factor (MET) gene mutation is a large class of mutations commonly seen in non-small cell lung cancer (NSCLC). MET mutation includes subtypes such as MET exon 14 skipping mutation (METex14m) and MET amplification (METamp). For advanced NSCLC with METex14m, Savolitinib has a high sensitivity as a member of tyrosine kinase inhibitors (TKIs). METamp is a relatively rare genetic mutation type which can serve as a driver gene to mediate primary and later acquired drug resistance of epidermal growth factor receptor (EGFR)-TKIs. For advanced NSCLC with secondary METamp, EGFR-TKIs combined with MET-TKIs are usually used in clinical treatment, while the optimal treatment strategy for advanced NSCLC with primary METamp has not yet been determined. For locally advanced NSCLC patients with positive driver gene mutations such as EGFR, anaplastic lymphoma kinase (ALK) fusion and METex14m, there have been relevant cases reported that neoadjuvant targeted therapy could achieve a good prognosis, but there have been no cases of neoadjuvant targeted therapy for locally advanced NSCLC patients with METamp. This report describes a case of a locally advanced NSCLC patient with dual driver gene mutations (EGFR L858R combined with primary METamp), the tumor did not shrink after 1 month of Gefitinib monotherapy, but significantly subsided after 4 months of Savolitinib monotherapy. After radical surgery, the pathological results proved pathological complete response (pCR) of the tumor, and the patient had a good response to postoperative continual Savolitinib treatment, with no recurrence nor metastasis observed to date. This case reports the feasibility and effectiveness of neoadjuvant targeted therapy for locally advanced NSCLC with primary METamp, aiming to provide effective reference for perioperative treatment of locally advanced NSCLC with primary METamp.

浸润性腺癌是常见的非小细胞肺癌（non-small cell lung cancer, NSCLC）病理类型之一，具有较高的驱动基因突变发生率，如表皮生长因子受体（epidermal growth factor receptor, EGFR）、间变性淋巴瘤激酶（anaplastic lymphoma kinase, ALK）、间质-上皮细胞转化因子（mesenchymal-epithelial transition factor, MET）、鼠类肉瘤病毒癌基因（Kirsten rat sarcoma viral oncogene, KRAS）等基因突变。MET基因突变包括MET外显子14跳跃突变（MET exon 14 skipping mutation, METex14m）、MET扩增（MET amplification, METamp）以及罕见的MET融合突变等亚型^[1,2]^。METex14m在肺腺癌中的发生率为2.8%，在肺肉瘤样癌中的发生率可高达22%，且一般不与EGFR、KRAS、ALK融合等其他敏感基因突变共存^[1]^。METamp也是一种独立的致癌驱动基因，在肺腺癌中的发生率约为1.7%，可以介导针对EGFR-酪氨酸激酶抑制剂（tyrosine kinase inhibitors, TKIs）的原发性耐药和后期药物获得性耐药^[3,4]^。2021年6月，赛沃替尼（Savolitinib）在中国被批准用于治疗对化疗不耐受或经过铂类化疗后疾病进展的具有METex14m的晚期NSCLC患者，然而赛沃替尼对于携带METamp的NSCLC的作用效果仍存在较大不确定性^[5,6]^。

临床中有接近30%的NSCLC患者在疾病诊断时为局部晚期（IIIA/IIIB期），这部分患者直接行手术治疗的预后效果较差，往往推荐新辅助治疗诱导肿瘤降期后再行根治性手术^[7]^。传统的新辅助化疗对局部晚期NSCLC患者整体预后的改善极为有限，而随着免疫治疗的发展，对于驱动基因突变阴性的局部晚期NSCLC患者，新辅助化疗联合免疫治疗已逐步证实可以获得比新辅助化疗更好的预后^[7]^。对于驱动基因突变阳性的局部晚期NSCLC患者，新辅助化疗联合免疫治疗的效果并不确定，新辅助靶向治疗是值得研究的方向^[8]^。有病例报道了具有EGFR突变、ALK融合突变和METex14m突变的局部晚期NSCLC患者接受新辅助靶向治疗的可行性^[9⇓-11]^，但目前尚未有伴随METamp突变的局部晚期NSCLC患者进行新辅助靶向治疗的案例。本文报道了1例携带EGFR L858R和METamp双驱动基因突变的局部晚期NSCLC患者，在接受吉非替尼短期治疗无效后，又接受了赛沃替尼诱导治疗并获得影像学明显缓解后进行了根治性手术，术后病理确认肿瘤发生病理学完全缓解（pathological complete response, pCR），为未来进一步探索具有METamp突变NSCLC的最佳治疗方案、探索新辅助靶向治疗提供了重要参考依据。

## 1 病例报道

患者，男性，70岁，主因“乏力、消瘦3个月”就诊。患者长期吸烟史40余年，每天约20支，并患有慢性阻塞性肺病（chronic obstructive pulmonary disease, COPD）10余年。该患者因长年未控制的COPD导致肺功能严重下降，无法进行大部分日常活动，入院时体力状态（performance status, PS）评分为2分。胸部增强计算机断层扫描（computed tomography, CT）显示左肺下叶软组织肿块影，范围约6.0 cm×5.0 cm，局部侵犯下肺静脉及下叶支气管，紧贴胸主动脉左侧壁，但胸腔内并无明显肺门及纵隔区域淋巴结肿大（图1A、1B）。癌胚抗原等肿瘤标志物指标、颅脑磁共振成像和全身骨扫描等其他检查未发现异常，初诊时的肿瘤临床分期为IIIA期（cT4N0M0）。CT引导下肺肿物细针穿刺活检病理确诊为浸润性肺腺癌，Ki-67指数为40%。同时采取穿刺获取的肺癌组织行基因检测显示存在EGFR L858R和METamp共突变，其中EGFR L858R的突变丰度为11.1%，METamp的基因拷贝数为4.14。肿瘤组织程序性细胞死亡配体1（programmed cell death ligand 1, PD-L1）高度表达[肿瘤细胞阳性比例分数（tumor proportion score, TPS）为90%]。

**图1:**
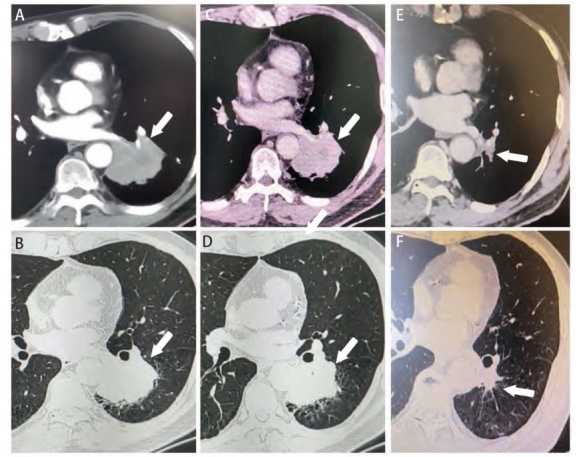
患者靶向治疗前后影像学资料。A：开始治疗前胸部CT肺窗肿瘤特征；B：开始治疗前胸部CT纵隔窗肿瘤特征；C：吉非替尼治疗后胸部CT肺窗肿瘤特征；D：吉非替尼治疗后胸部CT纵隔窗肿瘤特征；E：赛沃替尼治疗后胸部CT肺窗肿瘤特征；F：赛沃替尼治疗后胸部CT纵隔窗肿瘤特征。图中箭头指左肺下叶肿瘤病灶。

患者初诊时因PS评分较差，肺通气功能严重障碍，存在肺癌根治性手术的相对禁忌证，经多学科诊疗（multi-disciplinary treatment, MDT）后建议行同步放化疗。但由于不可抗力因素（期间遭遇新冠肺炎隔离措施），患者最终未能于放疗科住院治疗，且随访也暂时中断。此后患者于16周后返院并提供既往治疗经过：患者首先于院外自行购买吉非替尼（250 mg qd）治疗4周，于当地复查胸部CT显示肿瘤无明显缩小（图1C、1D）。随后又自行停用吉非替尼，院外购买赛沃替尼口服（600 mg qd），治疗12周后再次复查胸部CT提示左下肺门区域肿瘤明显缩小，边界不清，范围约2.8 cm×2.3 cm，且对主动脉弓的包绕范围明显缩小（图1E、1F）。治疗期间，患者仅出现轻度皮疹、腹泻等症状，没有出现严重不良反应，且得益于戒烟和呼吸内科治疗，期间肺功能也有明显改善，PS评分提高至1分。

患者住院后再次进行MDT评估后，于2022年8月11日接受了开胸左肺下叶切除+淋巴结清扫术（肺癌根治术）。整体手术过程顺利，患者术后无感染、出血等并发症。患者术后恢复良好，5 d后出院，无任何并发症。术后石蜡病理结果肉眼所见显示：送检左肺下叶组织标本，大小为19.0 cm×10.0 cm×4.0 cm，支气管断端直径2.5 cm，距支气管断端1.0 cm，支气管黏膜内见一肿物，大小为3.5 cm×3.0 cm×2.5 cm，肿物切面灰白色，质地硬，可见较多纤维化，肿瘤周围全部取材。镜下所见显示：肿瘤床内未见残留肿瘤细胞，可见淋巴细胞、胆固醇结晶、泡沫细胞聚集（图2）。且支气管端阴性，区域淋巴结未见转移，分组如下：4区0/3，5区0/1，7区0/3，9区0/2，10区0/1，11区0/3，12区0/4，13区0/4。因此，基于肿瘤主体病灶全部取材结果和清扫淋巴结病理结果，评估肿瘤达到pCR。患者术后持续使用赛沃替尼，未进行放化疗等其他治疗，并继续进行规律随访，至今未发生肿瘤复发及3/4级不良事件（图3）。

**图2:**
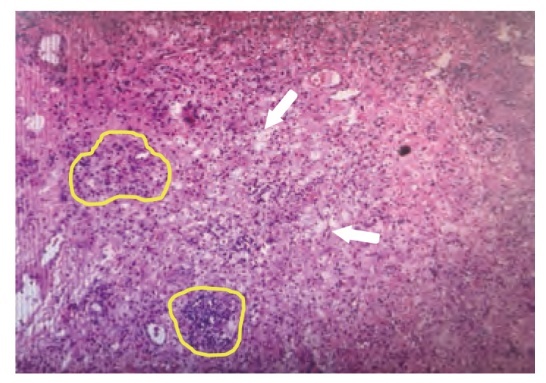
切除肿瘤的HE染色结果显示瘤床内未见残留肿瘤细胞（×40）。箭头指间质中泡沫细胞，黄色虚线圈内指聚集的淋巴细胞。

**图3:**
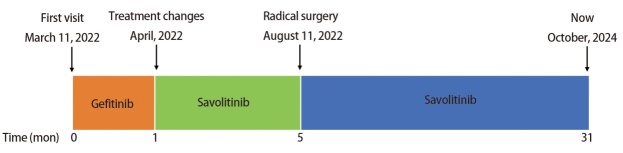
患者接受靶向治疗全过程时间线

## 2 讨论

MET基因突变是NSCLC中一类重要的癌症驱动基因及治疗靶点，其包括多种形式，其中METex14m和METamp较为多见。赛沃替尼等MET-TKIs已经在国内获批用于伴随METex14m突变的晚期NSCLC患者，而METamp多作为EGFR突变的原发或继发性耐药基因突变而存在，目前尚没有足够的证据支持MET-TKIs单药治疗对伴随METamp突变NSCLC的疗效^[12]^。本病例的特殊之处在于，患者在初诊时经MDT评估为不适合手术治疗的局部晚期NSCLC，但患者在初始治疗阶段并没有接受标准的同步放化疗，而是自主选择了副作用更小、用药方式更便捷的靶向治疗，基于EGFR L858R突变的存在，患者首先选择的一线治疗方案为吉非替尼，此后因吉非替尼疗效欠佳而自行改为赛沃替尼二线治疗。出人意料的是，患者经过赛沃替尼单药治疗诱导后获得肿瘤的明显影像学缓解，而肺癌根治性切除术后的病理结果进一步提示术前新辅助赛沃替尼靶向治疗诱导肿瘤发生了pCR，显示出赛沃替尼单药治疗对于伴随METamp突变NSCLC的价值，以及赛沃替尼在伴随METamp的局部晚期NSCLC新辅助治疗中具有一定安全性和有效性。

METamp包括原发性METamp和继发性METamp。继发性METamp一般是EGFR突变的晚期NSCLC患者经过长时间EGFR-TKIs治疗之后所出现的一种耐药基因突变形式。METamp在EGFR突变NSCLC中的作用在体外筛选的耐药细胞系中被发现，随后被确立为EGFR-TKIs耐药的临床分子标记^[12]^。随着奥希替尼作为EGFR突变晚期NSCLC患者的一线治疗方案的增加以及EGFR T790M突变介导的EGFR-TKIs耐药发生率的降低，METamp介导的EGFR-TKIs获得性耐药的比例逐渐增加，这也是EGFR-TKIs耐药的保守机制^[13]^。针对继发性METamp的情况，临床中一般是在最初的EGFR-TKIs治疗中加入MET-TKIs来解决^[12,13]^。然而，两种靶向药物联合使用会导致毒性增加，患者经常需要中断治疗或减少剂量，这可能会限制治疗效果^[5]^。原发性METamp指的是METamp也可以作为主要或共同驱动基因突变介导NSCLC的发生发展。METamp作为主要驱动基因突变，在1%-5%的初始治疗NSCLC患者中出现；作为一种共同驱动基因突变，它出现在2%-10%的EGFR突变初始治疗NSCLC患者中^[13]^。有研究^[5]^表明，原发性METamp作为EGFR突变的共同驱动基因突变，与晚期NSCLC患者一线EGFR-TKIs单药治疗后预后不良显著相关，而EGFR-TKIs联合赛沃替尼具有一定效果。Ma等^[14]^描述了1例具有EGFR L858R突变和原发METamp共突变的初诊晚期肺腺癌患者，一线治疗采取奥希替尼联合贝伐珠单抗和含铂化疗出现原发性耐药，之后开始赛沃替尼单药治疗有效，最后一次随访临床评价接近完全缓解，无进展生存期超过7个月。这些病例说明METamp可能在这种共同驱动基因突变中处于主导地位，是导致原发性EGFR-TKIs耐药的原因，且对赛沃替尼单药治疗具有良好的敏感性。

目前针对局部晚期NSCLC进行新辅助靶向治疗的研究进展较为缓慢。neoADAURA研究提示EGFR敏感突变NSCLC患者行新辅助靶向奥希替尼治疗可观察到无病生存期（disease-free survival, DFS）获益^[10]^。对于其他基因突变，如ALK融合突变甚至METex14m，也有新辅助靶向治疗获得明显病理学缓解的报道^[9,11,15]^，但针对METamp的局部晚期NSCLC，尚未有新辅助靶向治疗的病例报道。本病例提示赛沃替尼单药新辅助治疗在原发性METamp突变局部晚期NSCLC中是可行的，而与新辅助化疗相比，赛沃替尼新辅助靶向治疗在肿瘤治疗效果和患者生活质量上都有一定优势。

需要注意的是，本病例中患者采取的赛沃替尼属于二线治疗，一线治疗既没有选择更有效的EGFR-TKIs奥希替尼，也没有选择EGFR-TKIs与MET-TKIs联合治疗，因此一线使用赛沃替尼单药治疗作为新辅助诱导治疗是否能达到同样的pCR效果尚不清楚。另外，针对MET突变的新辅助靶向治疗的最佳持续时间也尚无定论。Zhang等^[15]^提到1例伴随METex14m的IIIA期NSCLC患者接受赛沃替尼新辅助治疗4周后肿瘤明显缩小，但术后病理证实肿瘤未达到pCR且仍旧存在淋巴结转移。Fu等^[11]^对1例伴随METex14m的IIIA期NSCLC患者采取赛沃替尼新辅助治疗5周的时间，同样也未达到pCR。本例患者术前赛沃替尼治疗时间为12周，肿瘤获得了pCR。这些病例表明，赛沃替尼新辅助治疗的效果与MET突变亚型、新辅助治疗持续时间均有一定关联。

综上所述，本病例总结了1例具有EGFR L858R和METamp共同驱动基因突变的局部晚期NSCLC患者先后进行吉非替尼、赛沃替尼单药治疗的效果，不仅揭示了METamp在EGFR-TKIs原发性耐药中的作用，还首次验证了赛沃替尼单药治疗在原发METamp突变局部晚期NSCLC新辅助靶向治疗中的疗效，为METamp突变局部晚期NSCLC的围手术期治疗提供了一种潜在更优的治疗选择。METamp参与EGFR-TKIs原发性耐药的机制值得未来进一步探索，未来需要更多的临床试验提供更有力的证据来评估赛沃替尼在MET突变NSCLC新辅助治疗中的结局和长期预后。
